# Preface

**DOI:** 10.1093/jacamr/dlab075

**Published:** 2021-06-15

**Authors:** Dilip Nathwani, Conor Jamieson, Trisha Peel, Pilar Retamar-Gentil

As this is the first Supplement produced by *JAC-Antimicrobial Resistance* (*JAC-AMR*) we thought it would be helpful for readers for us to briefly outline our vision and intention for these Supplements.

*JAC-AMR* is a unique, open access, peer-reviewed journal that incorporates traditional peer-reviewed research articles (including opinions, reviews and original articles) and educational resources (peer reviewed and non-peer reviewed). **Research** articles are creative and systematic work undertaken to increase the stock of knowledge. The research submitted may be used to develop further knowledge on a topic, or for education. **Education** resources, which may use explicit or tacit knowledge, are those that aim to facilitate learning or the acquisition of knowledge, skills, and beliefs. Both aim to improve practice. Educational resources may include typical teaching/training videos, presentations, discussions and storytelling. We believe this mixed approach to learning will allow sharing of a broader range of styles and modes of learning resource. We hope you that you enjoy this new format as we continue to innovate concerning how we learn and share knowledge.

This Supplement offers readers knowledge in the form of research and evolving educational information and experience relating to the clinical role of cefiderocol, a novel siderophore cephalosporin antibiotic, in the treatment of MDR Gram-negative infections.

The Supplement consists of two research articles: a review that provides a detailed overview of antimicrobial resistance during and beyond COVID-19 (Professor David Livermore)^1^ and a case report (Dr Mabayoje and colleagues)^2^ of the compassionate use of cefiderocol for the treatment of *Acinetobacter baumannii* prosthetic joint infection. This case report is also accompanied by an educational 7 min PowerPoint video presentation of the case by one of the authors, Dr David Wareham. Both of these articles were subject to peer and editorial review and are presented as traditional journal articles.

In addition to the research articles, there are four education resources that take the form primarily of narrated PowerPoint presentation videos with accompanying transcripts. All these resources were subject to editorial review and full transparency declarations.

The Supplement is introduced by Dr Abid Hussain.^3^ He outlines the novel mechanism of action of cefiderocol, its activity and stability against a range of carbapenemases, and the changing epidemiology of Gram-negative bacterial infections, with a focus on the UK, and highlights the approval for use of cefiderocol in Europe combined with the introduction of EUCAST breakpoints and guidance to support laboratories. In the second education video,^4^ Dr Karas, from Shionogi, expands on the mechanism of action and activity of cefiderocol. Importantly, he provides a review of the principal points from the analysis of key clinical trial data for cefiderocol as well as outcomes across a range of infections, populations and settings. He goes on to summarize the core *in vitro* data, highlighting the universal *in vitro* activity of cefiderocol against all WHO priority 1 critical pathogens, and the real-world-setting studies of the use of the agent since 2016. The presentation includes an interesting discussion of the challenges associated with how susceptibility testing for this agent is currently performed. This is followed by a compelling 15 min PowerPoint discussion by Professor Edgeworth, a microbiologist from the UK. He provides a personal critical analysis of the data provided by Dr Karas with a particular focus on the interpretation of data from one key efficacy study: CREDIBLE-CR. Instructively, he shares a previously published case study of a complex severe infection (native valve aortic endocarditis due to an MDR *Pseudomonas aeruginosa*) as a means of highlighting the real-world challenges of susceptibility testing for new agents and evaluating their real-world use and impact on clinical care and outcomes.

Recognizing there is much to be learnt from sharing clinical experiences, a common occurrence in everyday clinical practice, we include two clinical examples. Dr Chavda and colleagues^5^ provide a written example of compassionate use of cefiderocol in a patient with severe osteomyelitis caused by XDR *Pseudomonas aeruginosa*. This is also presented by Mr Mark Gilchrist in a PowerPoint video presentation. The second—the case report and accompanying video mentioned previously^2^—details the compassionate use of cefiderocol for the treatment of *A. baumannii* prosthetic joint infection. Finally, Professor Falcone^6^ provides a condensed overview of the cefiderocol compassionate use case series previously published in *Clinical Infectious Diseases*,^7^ as well as his own unpublished experience in the ICU setting.

## References

[1] Livermore DM. Antibiotic resistance during and beyond COVID-19. JAC Antimicrob Resist 2021; 3 Suppl 1: i5–i16. 10.1093/jacamr/dlab052 PMC8210049 34223149

[2] Mabayoje DA, NicFhogartaigh C, Cherian BP et al Compassionate use of cefiderocol for carbapenem-resistant *Acinetobacter baumannii* prosthetic joint infection. JAC Antimicrob Resist 2021; 3 Suppl 1: i21–i24. 10.1093/jacamr/dlab055 PMC8251250 34223152

[3] Hussain A. *Education:* The role of the first siderophore cephalosporin Fetcroja^®^ (cefiderocol) in UK clinical practice: introduction. JAC Antimicrob Resist 2021; 3 Suppl 1: i3–i4. 10.1093/jacamr/dlab051 PMC8251259 34223148

[4] Karas JA, Edgeworth J. *Education:* An introduction to the core data for cefiderocol with reflections for a possible role within UK clinical practice. JAC Antimicrob Resist 2021; 3 Suppl 1: i17. 10.1093/jacamr/dlab053 PMC8251257 34223150

[5] Chavda A, Gilchrist M, Samarasinghe D. *Education:* A compassionate use of cefiderocol to treat osteomyelitis caused by an XDR *Pseudomonas aeruginosa*. JAC Antimicrob Resist 2021; 3 Suppl 1: i18–i20. 10.1093/jacamr/dlab054 PMC8251337 34223151

[6] Falcone M. *Education:* An overview from the author of ‘Cefiderocol as rescue therapy for *Acinetobacter baumannii* and other carbapenem-resistant Gram-negative infections in intensive care unit patients’. JAC Antimicrob Resist 2021; 3 Suppl 1: i25. 10.1093/jacamr/dlab056 PMC8251248 34223153

[7] Falcone M, Tiseo G, Nicastro M et al Cefiderocol as rescue therapy for *Acinetobacter baumannii* and other carbapenem-resistant Gram-negative infections in intensive care unit patients. Clin Infect Dis 2020; doi:10.1093/cid/ciaa1410. 10.1093/cid/ciaa1410 32941593

